# Plasma small ncRNA pair panels as novel biomarkers for early-stage lung adenocarcinoma screening

**DOI:** 10.1186/s12864-018-4862-z

**Published:** 2018-07-20

**Authors:** Yuhong Dou, Yong Zhu, Junmei Ai, Hankui Chen, Helu Liu, Jeffrey A. Borgia, Xiao Li, Fan Yang, Bin Jiang, Jun Wang, Youping Deng

**Affiliations:** 1 Department of Clinical Laboratory, Shenzhen Baoan Shajing People’s Hospital, Guangzhou Medical University, Shenzhen, 518104 China; 2 Department of Anatomy and Cell Biology, Rush University Medical Center, Chicago, IL 60612 USA; 3 National Center of Colorectal Disease, Nanjing Municipal Hospital of Chinese Medicine, The Third Affiliated Hospital, Nanjing University of Chinese Medicine, Nanjing, 210001 China; 4 Department of Pathology, Rush University Medical Center, Chicago, IL 60612 USA; 5 Department of Thoracic Surgery, Peking University People’s Hospital, Beijing, 100044 China; 6 Department of Laboratory Medicine, Shiyan Taihe Hospital, College of Biomedical Engineering, Hubei University of Medicine, Shiyan, Hubei 442000 People’s Republic of China; 7 Bioinformatics Core, Department of Complementary & Integrative Medicine, University of Hawaii John A. Burns School of Medicine, Honolulu, HI 96813 USA

**Keywords:** Small non-coding RNA, Cancer screening, Biomarkers, Lung cancer

## Abstract

**Background:**

Lung cancer is a major cause of cancer-related mortality worldwide, and around two-thirds of patients have metastasis at diagnosis. Thus, detecting lung cancer at an early stage could reduce mortality. Aberrant levels of circulating small non-coding RNAs (small ncRNAs) are potential diagnostic or prognostic markers for lung cancer. We aimed to identify plasma small ncRNA pairs that could be used for early screening and detection of lung adenocarcinoma (LAC).

**Results:**

A panel of seven small ncRNA pair ratios could differentiate patients with LAC or benign lung disease from high-risk controls with an area under the curve (AUC) of 100.0%, a sensitivity of 100.0% and a specificity of 100.0% at the training stage (which included 50 patients with early-stage LAC, 35 patients with benign diseases and 29 high-risk controls) and an AUC of 90.2%, a sensitivity of 91.5% and a specificity of 80.4% at the validation stage (which included 44 patients with early-stage LAC, 32 patients with benign diseases and 51 high-risk controls). The same panel could distinguish LAC from high-risk controls with an AUC of 100.0%, a sensitivity of 100.0% and a specificity of 100.0% at the training stage and an AUC of 89.5%, a sensitivity of 85.4% and a specificity of 83.3% at the validation stage. Another panel of five small ncRNA pair ratios (different from the first) was able to differentiate LAC from benign disease with an AUC of 82.0%, a sensitivity of 81.1% and a specificity of 78.1% in the training cohort and an AUC of 74.2%, a sensitivity of 70.4% and a specificity of 72.7% in the validation cohort.

**Conclusions:**

Several small ncRNA pair ratios were identified as markers capable of discerning patients with LAC from those with benign lesions or high-risk control individuals.

**Electronic supplementary material:**

The online version of this article (10.1186/s12864-018-4862-z) contains supplementary material, which is available to authorized users.

## Background

Lung cancer is one of the main causes of cancer-related deaths worldwide [1]. In the USA, the incidence of lung cancer was estimated to be the second highest among all cancers (224,390 new cases in 2016), and lung cancer was predicted to be the most important cause of cancer-related mortality (158,080 deaths in 2016) [2]. About 80% of all lung cancers are non-small cell lung cancers (NSCLC) [3], and the two most common NSCLCs are lung adenocarcinoma (LAC, about 50%) and squamous cell carcinoma (SqCC, about 30%) [4].

Detecting lung cancer at its early stages could reduce mortality rates by 10- to 50-fold [5], but about two-thirds of patients have metastasis at diagnosis. Low-dose computed tomography (LDCT) provides a non-invasive method to detect early-stage tumors, but the rate of false-positive diagnosis is high [6, 7]. Molecular biomarkers could represent a promising screening approach.

Small non-coding RNAs (small ncRNAs), including microRNAs (miRNAs), nucleolar RNAs and tRNAs, have been shown to repress or degrade specific transcripts involved in cell fate and proliferation, cell death, energy metabolism and tumorigenesis [8]. When circulating in plasma/serum, mature miRNAs form a miRNA-Argonaute-protein complex that ensures their stability [9]. Therefore, small ncRNAs can be measured non-invasively with remarkable stability and repeatability [10]. Thus, aberrant levels of circulating miRNAs could be potential diagnostic or prognostic markers in lung [11], colorectal [12], prostate [13] and breast [14, 15] cancers.

The normalization of data for plasma/serum small ncRNA levels measured using quantitative reverse transcription polymerase chain reaction (qRT-PCR) is challenging, and this is an obstacle to standardization of the measurements. For this reason, a ratio-based method is critical for the analysis of data regarding circulating small ncRNAs. Many researchers have chosen to ‘spike’ samples with a synthetic RNA sequence (like *C. elegans* miR-39 and miR-54, or plant miRNAs) in order to normalize qRT-PCR data for circulating miRNA levels [16–18]. However, synthetic miRNAs are not protected from endogenous RNase activity and are rapidly degraded [18, 19], and none have been established for quantification of miRNAs in the blood [20–23]. miR-16 is frequently used as a control [24], but elevated serum levels of miR-16 correlate with bone metastasis in patients with breast cancer [25]. To bypass the normalization issue, some studies have analyzed plasma miRNA values by looking at the reciprocal ratios of miRNAs in the same sample [26–28].

In the present study, ratios of miRNAs in the same sample were used to reduce experimental variation. Rather than directly comparing the level of a single small ncRNA between groups, the ratio of any two small ncRNAs was calculated for the same sample, and then the expression level ratio was compared between different groups. Since the two targets are simultaneously measured in the same sample under the same conditions, the relative expression level (calculated as a ratio) should reflect a true value for comparison between groups.

Therefore, the aim of the present research was to perform a small ncRNA profiling study using next generation sequencing to measure whole genome-level small ncRNAs in plasma specimens from patients with early LAC, patients with benign lung lesions and high-risk controls.

## Methods

### Patient cohorts

For the training cohort, 1250 patients were enrolled at the Cancer Center of Rush University Medical Center (RUMC, Chicago, IL, USA) from March 2004 to October 2010. Among these patients, a sub-cohort of 114 patients (including 50 patients with early-stage [stage I or II] LAC, 35 patients with benign disease, and 29 high-risk individuals without lung disease) was selected for this pilot study. These patients had been followed up for at least two years and their diagnosis had not changed during follow-up.

LAC was staged according to the TNM Classification of Malignant Tumours, 6th edition. The inclusion criteria were: 1) disease confined to the chest without evidence of distant metastases; 2) no preoperative chemotherapy or radiotherapy within 1 year of the initial blood sampling; 3) a minimum of 2 years of clinical follow-up data; and 4) Caucasian.

Patients with benign lesions included participants with a range of non-neoplastic pulmonary disorders (e.g. granulomas, hamartomas and inflammatory lesions) as suggested by LDCT screening. All participants with benign diseases and the high-risk individuals without lung disease were followed-up by annual LDCT and remained cancer-free for a minimum of 2 years.

For the validation stage, 127 individuals (including 44 patients with early-stage LAC, 32 patients with benign diseases and 51 individuals without lung disease) were recruited at the Lung Cancer Biospecimen Resource Network (LCBRN, University of Virginia, Charlottesville, VA, USA) between March 2014 and October 2014. Note that the 127 individuals in the validation cohort were not from the original 1250 individuals used for the training cohort.

The study was approved by the institutional review board of RUMC. All participants provided written informed consent. The training cohort was from RUMC, and the validation cohort was from the LCBRN. The study was conducted at the RUMC.

### Collection of plasma samples

The plasma samples were collected and processed according to a standard protocol commonly used in many laboratories. All blood samples were collected using EDTA-anticoagulant tubes and centrifuged first at 4000 rpm for 10 min and then at 12,000 rpm for 15-min to completely remove cell debris. The supernatant (plasma) was stored at − 80 °C until analysis. No vigorous shaking or mixing was allowed during the processing of the samples. All samples were collected when the diagnosis was first made.

### Experimental strategy

To obtain an expression profile of plasma small ncRNAs that was specific for LAC, initial screening by Illumina next-generation sequencing and validation by qRT-PCR were used on an individual basis. The first step was to compare the profiles of the plasma expression ratios of small ncRNAs between participants. Then, specific small ncRNAs were tested.

### RNA isolation, qRT-PCR and Illumina next-generation sequencing

RNA isolation was performed as described previously [29]. Total RNA, including miRNA, was isolated from plasma using the Qiagen miRNeasy Mini kit (Qiagen, Valencia, CA, USA) in accordance with the manufacturer’s protocol, with minor modifications. In brief, 0.5 mL of plasma was diluted 1:1 with RNase-free water (a total of 1 mL) to achieve full phase separation. QIAzol® LS Reagent (3 mL) was added to each sample. The sample (total of 4 mL) was mixed in a tube, vortexed for 10 s, and incubated at room temperature for 15 min to allow complete dissociation of the nucleoprotein complex. The homogenized solution was centrifuged at 12,000×g for 10 min at 4 °C. The supernatant was transferred, and 0.8 mL of chloroform was added. After mixing vigorously for 15 s, the sample was centrifuged at 12,000×g for 15 min. The upper aqueous phase was carefully transferred to a new collection tube, and 2.5× volume of ethanol was added. The sample was applied directly to a silica membrane, and the RNA was bound and cleaned with buffers provided by the manufacturer to remove impurities. The immobilized RNA was collected from the membrane with 16 μL of RNase-free water (pre-warmed at 80 °C).

Small ncRNAs were measured using TaqMan MicroRNA Reverse Transcription Kits (Applied Biosystems, Foster City, CA, USA) in accordance with the manufacturer’s protocol. Briefly, about 30 ng enriched RNA was reverse transcribed with a TaqMan MicroRNA Reverse Transcription Kit in a reaction volume of 15 μL. The expression levels of the small ncRNAs were quantified in triplicate by qRT-PCR using human TaqMan MicroRNA Assay Kits (Applied Biosystems) and an iPLEX 4 system (Eppendorf, Hauppauge, NY, USA).

Illumina next-generation sequencing was used according to a method described previously [30]. Small RNA sequencing (smRNA-seq) was first performed to identify plasma miRNAs and some other circulating small ncRNAs in six samples pooled from 29 high-risk healthy individuals (there were 30 samples originally, but technical failure occurred in case), 30 individuals with benign lesions and 30 patients with LAC. The samples were from the training cohort. The pooled samples were made using 500 μL from each individual. Around 20 million reads were undertaken per sample, and about 90% of the reads aligned to the human genome.

For the library preparation, 6-μL volumes of the eluates from the plasma RNA isolation were used. Library preparation was performed using a minor modification of the Illumina protocol (Illumina, San Diego, CA, USA). A miRNA library was made from each RNA sample by 3library was made from each RNA sample by human genome ligation, reverse transcription, and PCR amplification. Libraries were then pooled in batches of 12 samples of equal amounts and clustered with a concentration of 10.5 pmol in one lane for each single-read flow cell using cBot (Illumina). Sequencing (50 cycles) was performed on a HiSeq 2500 system (Illumina) using the primer sequences listed in Table 1. Demultiplexing of the raw sequencing data and generation of the FASTQ files were performed using CASAVA v1.8.2 (Illumina).

**Table 1:** Primer sequences of the small ncRNAs

Small ncRNA	Sequence (5′- > 3′)
hsa-miR-101-3p	TACAGTACTGTGATAACTGAAG
hsa-miR-126-5p	CATTATTACTTTTGGTACGCG
hsa-miR-152-3p	TCAGTGCATGACAGAACTTGG
hsa-miR-19a-3p	TGTGCAAATCTATGCAAAACTGA
hsa-miR-22-3p	AAGCTGCCAGTTGAAGAACTGT
hsa-miR-374a-5p	TTATAATACAACCTGATAAGTG
hsa-miR-378a-3p	ACTGGACTTGGAGTCAGAAGGC
hsa-miR-423-5p	TGAGGGGCAGAGAGCGAGACTTT
hsa-sno-SNORD119	ATTAATGATGAGATATAACCTTGACTGAAGCTGATGA
hsa-sno-U57	GGAGGTGATGAACTGTCTGAGCCTGACC
hsa-tRNA-Thr-ACG	GGCGCGGTGGCCAAGTGG

Analysis of the smRNA-seq data.

The 3′ sequencing adapter was removed from the FASTQ files by local alignment of the adapter to the sequenced reads. Cutadapt software was used to remove the 3′ adapter [31]. All sequences having a length < 15 bp after adapter removal were discarded.

The reads in each library were summarized to tags in a quantified FASTA format. The FASTA reads were then mapped to the genome under consideration with Bowtie [32, 33]. To eliminate the ambiguous mapping hits, only the uniquely mapped loci with the fewest alignment mismatches were reported, allowing for a maximum of two mismatches [34–36]. The clean reads were then re-mapped back to human small ncRNAs using Bow-tie, the small ncRNA abundance was determined using Cufflinks software, and the annotation for each mapped locus was derived from ncRNA databases such as miRBase and Dfam [37, 38].

### Selection of differentially expressed small ncRNA pairs

To explore the high-throughput smRNA-seq data generated for each pooled sample, multiple-step bioinformatics data analysis was performed including adapter trimming, quantification, alignment, and identification of miRNAs and other small ncRNA species. Five types of small ncRNA were identified, including miRNAs (mature miRNAs and pre-miRNAs), snoRNAs, tRNAs, rRNAs and scRNAs. The averaged detectable numbers of small ncRNAs per pooled sample were narrowed down, based on at least 50 copies for a small ncRNA in any one of the pooled samples. Next, the ratios of any two small ncRNAs (except pre-miRNAs) were calculated in the same sample for all pooled samples, achieving on average about 333,336 ratios for each sample.

To provide a list of differentially expressed small ncRNA pairs, differential expression analysis was performed with comparison of LAC and benign diseases vs. control (i.e. individuals without lung disease), LAC vs. control, and LAC vs. benign, based on a fold change ≥2 and corrected *P*-value ≤0.05.

Using this strategy, a list of apparent small ncRNA pairs that fulfilled all three criteria (≥50 copies, fold change ≥2 and corrected P-value ≤0.05) was obtained from the sequenced samples, and these small ncRNA pairs were considered as candidate plasma biomarkers for LAC (Additional file 1: Table S1).

To demonstrate that the selected candidates were not only clinically useful and applicable but also highly sensitive, specific and accurate for the differentiation of LAC from benign disease and no lung disease (i.e. controls), receiver-operating characteristic (ROC) curve analysis was performed and the small ncRNA pairs were selected as individual plasma small ncRNA pair biomarkers for the diagnosis of LAC if they met these criteria: 1) sensitivity > 80%; 2) specificity > 80%; and 3) area under the ROC curve (AUC) > 0.800.

Data were compared in terms of lesion characteristics using WEKA 3.7 software (University of Waikato) for modeling [39]. Support vector machine recursive feature elimination (SVM-RFE) and a SVM classification algorithm were used to rank individual apparent small ncRNA pairs according to their predictive power to discriminate between the three groups in the training stage, and 10-fold cross validation was used to estimate the performance of the predictive model.

### Identification of a panel of small ncRNA pairs as candidate biomarkers for early-stage LAC using qRT-PCR

Small ncRNAs were measured in the training and validation cohorts using TaqMan MicroRNA Assay Kits (Applied Biosystems), in accordance with the manufacturer’s protocol. Briefly, about 30 ng of enriched RNA was reverse transcribed with a TaqMan Small ncRNA Reverse Transcription Kit (Applied Biosystems) in a 15-μL reaction volume. Expression levels of small ncRNAs were quantified in triplicate by qRT-PCR using human TaqMan MicroRNA Assay Kits (Applied Biosystems) and an iPLEX 4 system (Eppendorf). To bypass the normalization issue, we used the same ratio strategy described above to reduce experimental variation.

### Statistical and bioinformatics analysis

The analysis was performed using SPSS 20.0 (IBM, Armonk, NY, USA). After the plasma concentrations of the small ncRNAs had been log2-transformed, Student’s t-test was used to compare mean small ncRNA ratios between the LAC, benign and control groups. The difference between two groups (group X vs. group Y) in the plasma miRNA ratio was analyzed using the equation: RATIO_(group X vs. group Y)_ = mean of ΔCT_X_(miR1/miR2) – mean of ΔCT_Y_(miR1/miR2), where △CT_GROUP_(miR1/miR2) = CT_GROUP_(miR2) – CT_GROUP_(miR1). The fold change (FC) of group X/group Y was calculated as: FC = 2^RATIO^. The chi-squared test was used to compare the distributions of the training and validation cohorts with regard to gender, race and tumor stage. Two-sided *P*-values < 0.05 were considered statistically significant.

## Results

### Characteristics of the patients

There were no significant differences among the three groups in age, gender and smoking history (Table 2).

**Table 2:** Characteristics of the patients in the training and validation stages

	Training stage			Validation stage		
	LAC	Benign	Control	LAC	Benign	Control
	*n* = 50	*n* = 35	*n* = 29	*n* = 44	*n* = 32	*n* = 51
Age, years						
Mean	66.3	62.1	60.6	67.5	60.2	60.1
SD	7.9	9.2	8.1	10.7	14.5	7.5
Range	49–80	42–77	50–76	48–88	20–80	49–82
Gender, n (%)						
Male	21 (42.0)	18 (51.4)	13 (44.8)	20 (45.4)	17 (53.1)	25 (49.0)
Female	29 (58.0)	17 (48.6)	16 (55.2)	24 (54.6)	15 (46.9)	26 (51.0)
Smoking history, n (%)						
> 5 years	39 (78.1)	19 (54.3)	18 (62.1)	36 (81.8)	17 (53.1)	31 (60.8)
< 5 years	11 (21.9)	16 (45.7)	11 (37.9)	8 (18.2)	15 (46.9)	20 (39.2)
Tumor stage, n (%)						
Stage 0–1	28 (56.0)			26 (59.1)		
Stage 2	22 (44.0)			18 (40.9)		

There were no significant differences in age, gender and smoking history between groups. SD: standard deviation

Small ncRNA pairs that were differentially expressed between the three groups

**Fig. 1:**
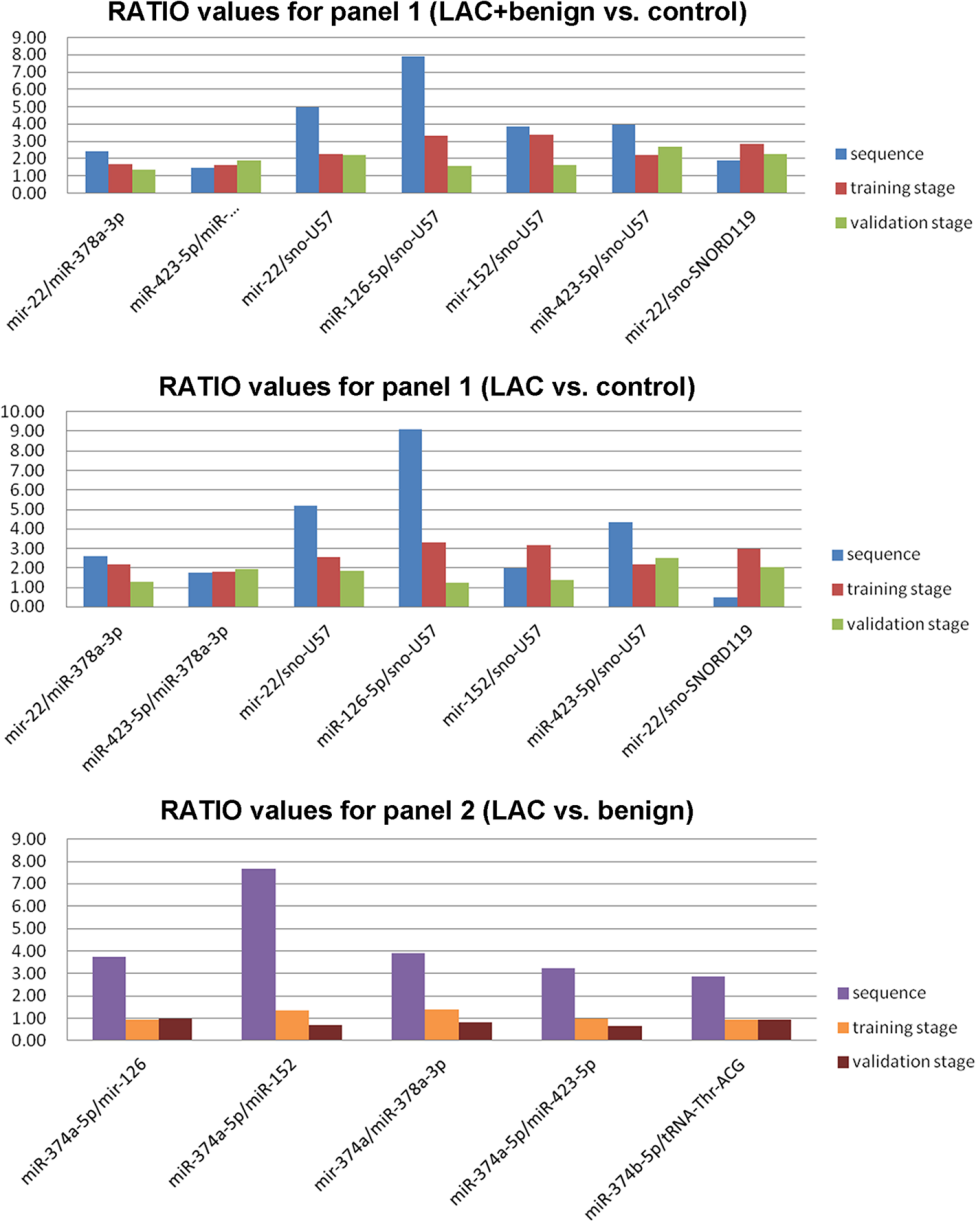
Comparison of RATIO values for two panels of ncRNA pairs between sequencing data and qRT-PCR data for the training and validation stages. Upper graph: panel 1, lung adenocarcinoma (LAC) and benign disease (benign) vs. no lung disease (control); middle graph: panel 1, LAC vs. control; lower graph: panel 2, LAC vs. benign

We identified 342 miRNAs, 47 tRNAs, 19 snoRNAs, 3rRNAs and 4 scrRNAs in the six pooled samples. The list of small ncRNA pairs that apparently fulfilled all three criteria in the training stage and were candidate biomarkers for LAC are listed in Additional file 1: Table S1. The ratios based on the sequencing data were found to be consistent with those from actual PCR data for the training and validation stages (Fig. 1). Data for each group describing the means and standard deviations for the expression ratios of the various small ncRNA pairs are presented in Additional file 1: Table S2. Furthermore, scatter plots comparing the expression ratio of each small ncRNA pair between groups are shown in Additional file 1: Figures S1–S3.

### A panel of small ncRNA pairs distinguished patients with LAC or benign disease from control individuals

**Fig. 2:**
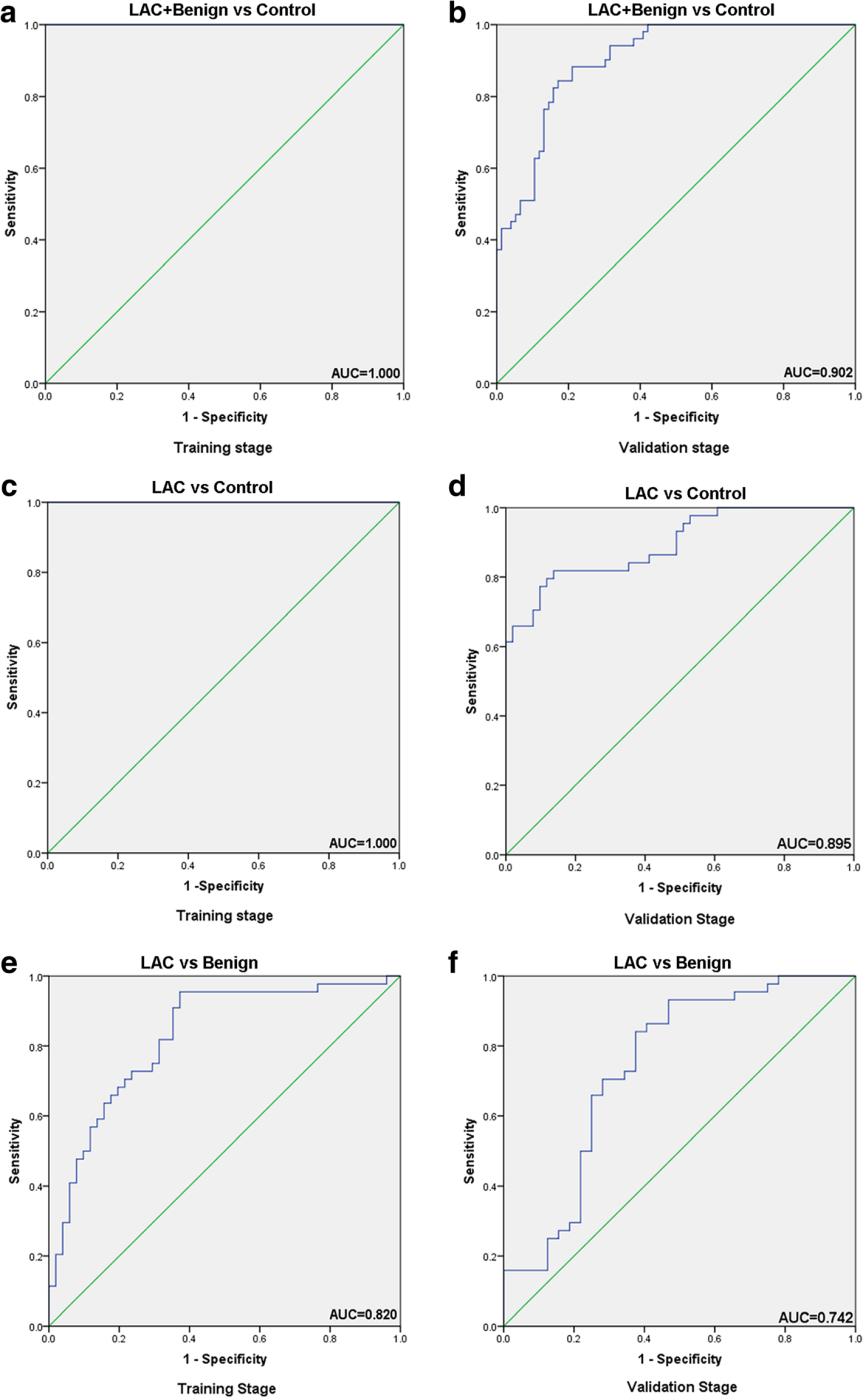
Receiver operating characteristic (ROC) curve analysis of small ncRNA pair panels for disease prediction in the training and validation stages. Shown are the area under the ROC curve (AUC) values of Panel 1 for lung adenocarcinoma (LAC) and benign vs. control (training: **a**; validation: **b**), Panel 1 for LAC vs. control (training: **c**; validation: **d**), and Panel 2 for LAC vs. benign (training: **e**; validation: **f**)

In the training stage, a panel of seven small ncRNA pairs (designated Panel 1) was identified as a candidate panel for differentiating patients with early-stage LAC or benign disease from controls; this panel included miR-22/miR-378, miR-423/miR-378, miR-22/sno-U57, miR-126/sno-U57, miR-152/sno-U57, miR-423/sno-U57 and miR-22/sno-DR119 (Table 3). All seven small ncRNA pairs showed significantly increased RATIO values in the LAC+benign group compared with the control group (Table 3). Analysis of the predictive power of this panel for the diagnosis of early-stage lung disease revealed an AUC of 100.0%, a sensitivity of 100.0% and a specificity of 100.0% in the training stage (Table 4 and Fig. 2a).

**Table 3:** Panels of small ncRNA pairs that distinguished between individuals with lung adenocarcinoma, benign lung disease and no lung disease (controls)

Small ncRNA pairs panels	Training stage						Validation stage					
	P-value	RATIO	FC	SEN	SPE	AUC	P-value	RATIO	FC	SEN	SPE	AUC
Panel 1 LAC+Benign vs. Control												
miR-22-3p/miR-378a-3p	1.35E-18	1.73	3.31	0.929	0.621	0.945	2.43E-08	1.37	2.58	0.882	0.588	0.795
miR-423-5p/miR-378a-3p	3.80E-08	1.61	3.05	0.953	0.552	0.849	3.92E-10	1.87	3.65	0.868	0.608	0.840
miR-22-3p/sno-U57	1.88E-18	2.31	4.97	0.953	0.828	0.950	1.71E-09	2.20	4.60	0.829	0.608	0.824
miR-126-5p/sno-U57	2.26E-24	3.31	9.91	0.953	0.862	0.984	4.47E-05	1.59	3.02	0.829	0.471	0.707
miR-152-3p/sno-U57	4.02E-21	3.37	10.32	0.941	0.828	0.970	2.48E-05	1.64	3.11	0.829	0.529	0.718
miR-423-5p/sno-U57	2.03E-12	2.19	4.57	0.929	0.621	0.896	1.71E-12	2.70	6.50	0.855	0.608	0.851
miR-22-3p/sno-SNORD119	1.77E-15	2.89	7.41	0.941	0.655	0.914	1.96E-08	2.24	4.72	0.855	0.510	0.782
Panel 1 LAC vs. Control												
miR-22-3p/miR-378a-3p	1.03E-23	2.18	4.54	0.960	0.966	0.992	2.15E-06	1.30	2.45	0.750	0.765	0.783
miR-423-5p/miR-378a-3p	4.20E-12	1.79	3.45	0.920	0.690	0.883	3.65E-09	1.94	3.84	0.727	0.706	0.845
miR-22-3p/sno-U57	1.58E-15	2.36	5.12	0.920	0.897	0.946	1.15E-06	1.84	3.58	0.773	0.725	0.816
miR-126-5p/sno-U57	2.66E-19	3.30	9.86	0.940	0.862	0.981	3.94E-03	1.24	2.36	0.636	0.667	0.679
miR-152-3p/sno-U57	2.83E-16	3.19	9.11	0.860	0.897	0.964	7.17E-04	1.40	2.63	0.636	0.686	0.708
miR-423-5p/sno-U57	5.04E-12	2.17	4.51	0.860	0.759	0.901	1.28E-09	2.49	5.61	0.750	0.765	0.853
miR-22-3p/sno-SNORD119	8.39E-14	3.07	8.38	0.880	0.724	0.931	2.03E-05	2.03	4.09	0.636	0.647	0.754
Panel 2 (LAC vs. Benign)												
miR-374a-5p/miR-126-5p	6.88E-03	0.93	1.90	0.820	0.429	0.667	2.02E-03	0.97	1.96	0.750	0.438	0.691
miR-374a-5p/miR-152-3p	1.39E-03	1.35	2.55	0.800	0.514	0.696	3.06E-02	0.70	1.63	0.750	0.313	0.625
miR-374a-5p/miR-378a-3p	9.93E-04	1.40	2.64	0.800	0.543	0.706	3.48E-02	0.82	1.76	0.864	0.313	0.618
miR-374a-5p/miR-423-5p	2.46E-02	0.96	1.94	0.820	0.314	0.622	4.26E-02	0.64	1.56	0.841	0.375	0.624
miR-374a-5p/tRNA-Thr-ACG	2.77E-02	0.94	1.91	0.760	0.229	0.680	2.09E-02	0.92	1.90	0.750	0.313	0.663

*AUC* area under receiver operating characteristic curve, *FC* fold change, *SEN* sensitivity, *SPE* specificity. RATIO and FC were calculated using the equations given in the Methods section

**Table 4:** Predictive values of small ncRNA pair panels at the training and validation stages

Small ncRNA pair panels	Sample size	SEN	SPE	PPV	NPV	FPR	FNR	AUC
Panel 1: LAC+Benign vs. Control								
Training	85 vs. 29	1.000	1.000	1.000	1.000	0.000	0.000	1.000
Validation	76 vs. 51	0.915	0.804	0.855	0.882	0.196	0.085	0.902
Panel 1: LAC vs. Control								
Training	50 vs. 29	1.000	1.000	1.000	1.000	0.000	0.000	1.000
Validation	44 vs. 51	0.854	0.833	0.795	0.882	0.167	0.146	0.895
Panel 2: LAC vs. Benign								
Training	50 vs. 35	0.811	0.781	0.860	0.714	0.219	0.189	0.820
Validation	44 vs. 32	0.704	0.727	0.864	0.500	0.273	0.296	0.742

*AUC* area under receiver operating characteristic curve, *FNR* false negative rate, *FPR* false positive rate, *LAC* lung adenocarcinoma, *NPV* negative predictive value, *PPV* positive predictive value, *SEN* sensitivity, *SPE* specificity

Panel 1 was further tested in the validation stage, which was independent of the training stage. The variations in the RATIO values of the seven small ncRNA pairs between groups were similar for the validation and training stages (Table 3). At the validation stage, the combination of these seven small ncRNA pair markers yielded a predictive power with a sensitivity of 84.3%, a specificity of 82.9% and an AUC of 90.2% (Table 4 and Fig. 2b).

As shown in Table 3, Panel 1 was able to distinguish the LAC group from the control group. All seven small ncRNA pairs had significantly higher RATIO values in the LAC group than in the control group (Table 3). The predictive power of Panel 1 for differentiating patients with early-stage LAC from controls had a sensitivity of 100.0%, a specificity of 100.0% and an AUC of 100.0% in the training stage (Table 4 and Fig. 2c) and a sensitivity of 81.8%, a specificity of 86.3% and an AUC of 89.5% in the validation stage (Table 4 and Fig. 2d).

### A specific panel of small ncRNA pair biomarkers distinguished LAC from benign disease

A panel of 5 small ncRNA pair markers (Panel 2) was found to specifically separate LAC from benign lesions; this panel included miR-374a-5p/miR-126-5p, miR-374a-5p/miR-152-3p, miR-374a-5p/miR-378a-3p, miR-374a-5p/miR-423-5p and miR-374a-5p/tRNA-Thr-ACG. All five small ncRNA pairs had a significantly higher RATIO value in the LAC group than in the benign group (Table 3). In the training stage, this panel demonstrated predictive power with a sensitivity of 81.1%, a specificity of 78.1% and an AUC of 82.0% (Table 4 and Fig. 2e). In the validation stage, the sensitivity was 70.4%, the specificity was 72.7%, and the AUC was 74.2% (Table 4 and Fig. 2f). Thus, the ability of Panel 2 to differentiate between the LAC and benign groups was not as good as the ability of Panel 1 to differentiate between the LAC and control groups.

## Discussion

In this present study, profiling of plasma small ncRNA pairs in patients with and without LAC identified a distinct panel of seven small ncRNA pairs that could help to predict LAC at an early stage. To the best of our knowledge, this is the first report using next generation sequencing of plasma small ncRNA pairs (other than miRNAs) for the early detection of lung cancer. Plasma is an ideal sample on which to base the development of a quick, non-invasive blood test for the early diagnosis of LAC. In the present study, the false positive rates for distinguishing lung disease (LAC and benign disease) from controls and LAC from controls were lower than those reported for LDCT screening alone (13–17.1%) [6, 7]. The sensitivity, specificity and AUC of these small ncRNA panels may not be high enough to readily distinguish between LAC, benign disease and controls using the profiles alone, but this study suggests that these small ncRNA panels could be used with LDCT-based screening methods to distinguish patients with LAC from high-risk individuals, potentially improving the currently available approaches [6, 7].

miR-22 suppresses lung cancer cell progression [40] and is a predictive marker for pemetrexed-based chemotherapy [41]. miR-126 inhibits NSCLC proliferation [42], enhances the sensitivity of NSCLC to anticancer agents [43] and is associated with the prognosis of NSCLC [44]. miR-152 regulates metastasis of NSCLC [45]. miR-374a suppresses lung cancer cell proliferation [46] and is a prognostic marker for NSCLC [47]. miR-378 is a tumor suppressor in NSCLC [48] but could be involved in brain metastasis [49]. The possible involvement of miR-423-5p in lung cancer has not been reported before.

The results of this study showed a sensitivity of 84.3%, specificity of 82.9% and AUC of 90.2% for distinguishing patients with lung disease (LAC or benign disease) from controls. In a previous investigation, a panel of 16 ratios involving 13 different miRNAs correctly classified 16 of 19 patients, with a sensitivity of 84% and a specificity of 80% [26]. Furthermore, a miRNA signature classifier algorithm showed a sensitivity of 87% and a specificity of 81% for the detection of lung cancer, and when this classifier algorithm was combined with LDCT, it reduced the false positive rate from 19.4 to 3.7% [27]. Other research showed that a 10-miRNA biomarker profile had high AUC, sensitivity and specificity values for the detection of NSCLC (97, 93 and 90%, respectively) [18]. A study that assessed miRNA in sputum samples identified four miRNAs that distinguished patients with LAC from control individuals with a sensitivity of 80.6% and a specificity of 91.7% [50].

The present study is not without limitations. The sample size was relatively small and the participants were from only two centers (one center for each cohort). SqCC samples were not included. Only Caucasians were included, limiting the generalizability of the results. A panel of small ncRNA pairs was not identified that could distinguish the LAC group from the benign and control groups (considered together rather than separately). Other RNAs, such as lncRNAs, ceRNAs and circRNAs, were not considered. Formal assessments of the internal and external reproducibility of the measurements were not performed. However, the present study did show a similar pattern of qRT-PCR results at the training and validation stages (which used independent cohorts), and repeat qRT-PCR experiments in the same samples 3 months after the initial measurements yielded consistent findings (data not shown). Additional studies are necessary to confirm the results of this study before this technique can be used as a screening method.

In the present study, the samples were prospectively collected from patients who had at least 2 years of clinical follow-up without a change in status. This should ensure that the data accurately reflect the disease status at the time of collection and means that we can potentially predict the cancer 2 years before it occurs. Because of the difficulties in normalizing the levels of small ncRNAs, the use of a ratio-based method for circulating small ncRNAs is probably key to identifying small ncRNA biomarkers, and this strategy will be validated in a larger dataset of individuals with no lung disease (controls), benign lung disease and lung cancer. If successfully validated, this ratio strategy could then be applied in the clinic setting, enabling the use of circulating small ncRNA biomarkers for the early detection of cancer in the future.

## Conclusions

Several small ncRNA pair ratios were identified as markers capable of discerning patients with LAC from those with benign lesions or high-risk control individuals.

## Additional files

Additional file 1 **Table S1.** All candidate ncRNA pairs for lung cancer prediction**. Table S2.** Mean and standard deviation values of the expression ratios of the various ncRNA pairs for each group. **Figure S1.** Scatter plots comparing the expression ratios of the seven small ncRNA pairs in Panel 1 between the LAC+benign group and the control group for the training and validation stages. **Figure S2.** Scatter plots comparing the expression ratios of the seven small ncRNA pairs in Panel 1 between the LAC group and the control group for the training and validation stages. **Figure S3.** Scatter plots comparing the expression ratios of the five small ncRNA pairs in Panel 2 between the LAC group and the benign group for the training and validation stages. (DOCX 557 kb)

## Abbreviations

(LAC): lung adenocarcinoma
(LCBRN): Lung Cancer Biospecimen Resource Network
(miRNAs): microRNAs
(NSCLC): non-small cell lung cancers
(qRT-PCR): quantitative reverse-transcription polymerase chain reaction
(RUMC): Rush University Medical Center
(small ncRNAs): small non-coding RNAs
(smRNA-seq): small RNA sequencing
