# Supplementary material for: Plasma small ncRNA pair panels as novel biomarkers for early-stage lung adenocarcinoma screening
Source: BMC Genomics. 2018 Jul 20;19:545. doi: 10.1186/s12864-018-4862-z (PMC6053820; doi:10.1186/s12864-018-4862-z)

**Supplementary Material**

**Supplementary Table 1.** All candidate ncRNA pairs for lung cancer prediction

|  | **ncRNA pairs panels** | **Training stage** | | | | **Validation stage** | | | | |
| --- | --- | --- | --- | --- | --- | --- | --- | --- | --- | --- |
|  |  | P-value | RATIO | FC | AUC | | P-value | RATIO | FC | AUC |
| **Sequence number** | **LAC+Benign vs. Control** |  |  |  | 1.000 | |  |  |  | 0.945 |
| 1 | miR-22-3p/miR-101-3p | 2.80E-06 | 0.79 | 1.73 | 0.801 | | 2.96E-03 | 0.65 | 1.57 | 0.664 |
| 2 | miR-101-3p/miR-378a-3p | 1.53E-05 | 0.93 | 1.91 | 0.744 | | 4.31E-04 | 0.71 | 1.64 | 0.726 |
| 3 | miR-126-5p/miR-378a-3p | 2.25E-32 | 2.72 | 6.60 | 0.993 | | 3.97E-03 | 0.76 | 1.70 | 0.654 |
| 4 | miR-152-3p/miR-378a-3p | 1.63E-35 | 2.78 | 6.89 | 1.000 | | 1.32E-03 | 0.80 | 1.74 | 0.679 |
| 5 | miR-19a-3p/miR-378a-3p | 3.00E-05 | 1.29 | 2.45 | 0.843 | | 7.96E-04 | 0.79 | 1.73 | 0.686 |
| 6 | miR-22-3p/miR-378a-3p | 1.35E-18 | 1.73 | 3.31 | 0.945 | | 2.43E-08 | 1.37 | 2.58 | 0.795 |
| 7 | miR-374a-5p/miR-378a-3p | 3.71E-08 | 1.83 | 3.57 | 0.775 | | 4.68E-03 | 0.87 | 1.83 | 0.646 |
| 8 | miR-423-5p/miR-378a-3p | 3.80E-08 | 1.61 | 3.05 | 0.849 | | 3.92E-10 | 1.87 | 3.65 | 0.840 |
| 9 | miR-126-5p/miR-3960 | 5.15E-20 | 2.39 | 5.25 | 0.957 | | 9.08E-03 | 0.88 | 1.84 | 0.640 |
| 10 | miR-22-3p/miR-3960 | 1.80E-05 | 1.33 | 2.51 | 0.783 | | 2.32E-06 | 1.48 | 2.80 | 0.749 |
| 11 | miR-22-3p/sno-SNORD119 | 1.77E-15 | 2.89 | 7.41 | 0.914 | | 1.96E-08 | 2.24 | 4.72 | 0.782 |
| 12 | miR-374a-5p/sno-SNORD119 | 1.17E-11 | 3.02 | 8.08 | 0.889 | | 3.89E-05 | 1.74 | 3.35 | 0.710 |
| 13 | miR-378a-3p/sno-SNORD119 | 1.63E-04 | 1.16 | 2.23 | 0.744 | | 7.54E-04 | 0.87 | 1.83 | 0.718 |
| 14 | miR-101-3p/sno-U57 | 1.42E-06 | 1.52 | 2.86 | 0.803 | | 1.76E-05 | 1.55 | 2.92 | 0.755 |
| 15 | miR-126-5p/sno-U57 | 2.26E-24 | 3.31 | 9.91 | 0.984 | | 4.47E-05 | 1.59 | 3.02 | 0.707 |
| 16 | miR-152-3p/sno-U57 | 4.02E-21 | 3.37 | 10.32 | 0.970 | | 2.48E-05 | 1.64 | 3.11 | 0.718 |
| 17 | miR-19a-3p/sno-U57 | 7.21E-08 | 1.88 | 3.69 | 0.860 | | 5.92E-06 | 1.62 | 3.08 | 0.752 |
| 18 | miR-22-3p/sno-U57 | 1.88E-18 | 2.31 | 4.97 | 0.950 | | 1.71E-09 | 2.20 | 4.60 | 0.824 |
| 19 | miR-374a-5p/sno-U57 | 2.59E-11 | 2.42 | 5.35 | 0.843 | | 2.70E-05 | 1.70 | 3.25 | 0.707 |
| 20 | miR-3960/sno-U57 | 2.21E-04 | 0.92 | 1.89 | 0.760 | | 3.09E-05 | 0.72 | 1.64 | 0.699 |
| 21 | miR-423-5p/sno-U57 | 2.03E-12 | 2.19 | 4.57 | 0.896 | | 1.71E-12 | 2.70 | 6.50 | 0.851 |
| 22 | tRNA-Thr-ACG/sno-U57 | 6.00E-28 | 4.14 | 17.64 | 0.989 | | 9.67E-03 | 0.71 | 1.64 | 0.634 |
|  | **LAC vs. Control** |  |  |  | 1.000 | |  |  |  | 1.000 |
| 1 | miR-22-3p/miR-101-3p | 6.30E-07 | 0.95 | 1.93 | 0.846 | | 9.19E-03 | 0.71 | 1.64 | 0.662 |
| 2 | miR-423-5p/miR-101-3p | 1.56E-03 | 0.77 | 1.70 | 0.730 | | 9.73E-05 | 1.36 | 2.56 | 0.736 |
| 3 | miR-101-3p/miR-378a-3p | 2.20E-05 | 1.02 | 2.03 | 0.760 | | 1.61E-02 | 0.58 | 1.50 | 0.702 |
| 4 | miR-126-5p/miR-378a-3p | 6.17E-31 | 2.92 | 7.55 | 1.000 | | 2.48E-02 | 0.70 | 1.62 | 0.651 |
| 5 | miR-152-3p/miR-378a-3p | 3.47E-27 | 2.80 | 6.99 | 1.000 | | 3.98E-03 | 0.85 | 1.80 | 0.698 |
| 6 | miR-19a-3p/miR-378a-3p | 5.08E-07 | 1.59 | 3.01 | 0.914 | | 3.51E-03 | 0.81 | 1.76 | 0.698 |
| 7 | miR-22-3p/miR-378a-3p | 1.03E-23 | 2.18 | 4.54 | 0.992 | | 2.15E-06 | 1.30 | 2.45 | 0.783 |
| 8 | miR-374a-5p/miR-378a-3p | 1.21E-09 | 2.41 | 5.31 | 0.877 | | 7.00E-04 | 1.22 | 2.32 | 0.690 |
| 9 | miR-423-5p/miR-378a-3p | 4.20E-12 | 1.79 | 3.45 | 0.883 | | 3.65E-09 | 1.94 | 3.84 | 0.845 |
| 10 | miR-152-3p/miR-3960 | 1.78E-14 | 2.46 | 5.51 | 0.954 | | 4.80E-02 | 0.73 | 1.65 | 0.623 |
| 11 | miR-19a-3p/miR-3960 | 1.91E-03 | 1.25 | 2.39 | 0.812 | | 4.40E-02 | 0.69 | 1.61 | 0.656 |
| 12 | miR-22-3p/miR-3960 | 6.83E-09 | 1.79 | 3.45 | 0.879 | | 5.58E-04 | 1.17 | 2.25 | 0.713 |
| 13 | miR-374a-5p/miR-3960 | 1.05E-05 | 2.07 | 4.20 | 0.782 | | 4.87E-03 | 1.09 | 2.13 | 0.661 |
| 14 | miR-423-5p/miR-3960 | 2.00E-08 | 1.45 | 2.73 | 0.833 | | 3.44E-07 | 1.82 | 3.52 | 0.776 |
| 15 | miR-126-5p/sno-SNORD119 | 8.61E-22 | 1.96 | 3.89 | 0.984 | | 3.77E-03 | 1.43 | 2.70 | 0.703 |
| 16 | miR-152-3p/sno-SNORD119 | 1.94E-17 | 1.84 | 3.59 | 0.949 | | 1.14E-03 | 1.59 | 3.00 | 0.718 |
| 17 | miR-19a-3p/sno-SNORD119 | 4.94E-02 | 0.63 | 1.55 | 0.742 | | 2.48E-03 | 1.55 | 2.92 | 0.719 |
| 18 | miR-22-3p/sno-SNORD119 | 8.39E-14 | 3.07 | 8.38 | 0.931 | | 2.03E-05 | 2.03 | 4.09 | 0.754 |
| 19 | miR-374a-5p/sno-SNORD119 | 8.24E-11 | 3.24 | 9.44 | 0.902 | | 9.13E-05 | 1.95 | 3.87 | 0.728 |
| 20 | miR-378a-3p/sno-SNORD119 | 2.58E-03 | 0.80 | 1.74 | 0.710 | | 1.56E-02 | 0.74 | 1.67 | 0.712 |
| 21 | miR-423-5p/sno-SNORD119 | 4.95E-04 | 0.83 | 1.78 | 0.702 | | 2.32E-07 | 2.68 | 6.40 | 0.798 |
| 22 | miR-101-3p/sno-U57 | 2.74E-05 | 1.40 | 2.64 | 0.790 | | 3.71E-03 | 1.13 | 2.19 | 0.722 |
| 23 | miR-126-5p/sno-U57 | 2.66E-19 | 3.30 | 9.86 | 0.981 | | 3.94E-03 | 1.24 | 2.36 | 0.679 |
| 24 | miR-152-3p/sno-U57 | 2.83E-16 | 3.19 | 9.11 | 0.964 | | 7.17E-04 | 1.40 | 2.63 | 0.708 |
| 25 | miR-19a-3p/sno-U57 | 1.66E-06 | 1.98 | 3.94 | 0.866 | | 7.52E-04 | 1.35 | 2.56 | 0.732 |
| 26 | miR-22-3p/sno-U57 | 1.58E-15 | 2.36 | 5.12 | 0.946 | | 1.15E-06 | 1.84 | 3.58 | 0.816 |
| 27 | miR-374a-5p/sno-U57 | 4.97E-09 | 2.80 | 6.95 | 0.871 | | 4.22E-05 | 1.76 | 3.38 | 0.730 |
| 28 | miR-3960/sno-U57 | 4.27E-03 | 0.72 | 1.65 | 0.739 | | 4.05E-04 | 0.67 | 1.59 | 0.687 |
| 29 | miR-423-5p/sno-U57 | 5.04E-12 | 2.17 | 4.51 | 0.901 | | 1.28E-09 | 2.49 | 5.61 | 0.853 |
|  | **LAC vs. Benign** |  |  |  | 0.754 | |  |  |  | 0.742 |
| 1 | miR-374a-5p/miR-101-3p | 7.51E-03 | 1.19 | 2.28 | 0.676 | | 7.49E-03 | 1.13 | 2.19 | 0.672 |
| 2 | miR-374a-5p/miR-126-5p | 6.88E-03 | 0.93 | 1.90 | 0.667 | | 2.02E-03 | 0.97 | 1.96 | 0.691 |
| 3 | miR-374a-5p/miR-152-3p | 1.39E-03 | 1.35 | 2.55 | 0.696 | | 3.06E-02 | 0.70 | 1.63 | 0.625 |
| 4 | miR-374a-5p/miR-378a-3p | 9.93E-04 | 1.40 | 2.64 | 0.706 | | 3.48E-02 | 0.82 | 1.76 | 0.618 |
| 5 | miR-374a-5p/miR-423-5p | 2.46E-02 | 0.96 | 1.94 | 0.622 | | 4.26E-02 | 0.64 | 1.56 | 0.624 |
| 6 | miR-374a-5p/tRNA-Thr-ACG | 2.77E-02 | 0.94 | 1.91 | 0.680 | | 2.09E-02 | 0.92 | 1.90 | 0.663 |

LAC: lung adenocarcinoma; FC: fold change; AUC: area under curve.

**Supplementary Table 2.** Mean and standard deviation values of the expression ratios of the various ncRNA pairs for each group.

| **Sequence number** | **ncRNA pairs panels** | **Group** | **Training stage** | | | **Validation stage** | | |
| --- | --- | --- | --- | --- | --- | --- | --- | --- |
|  |  |  | No. of samples | Mean | SD | No. of samples | Mean | SD |
|  | **LAC+Benign vs. Control** |  |  |  |  |  |  |  |
| 1 | miR-22-3p/miR-378a-3p | LAC+Benign | 85 | 3.63 | 0.80 | 76 | 4.25 | 1.08 |
| 2 | miR-22-3p/miR-378a-3p | Control | 29 | 1.91 | 0.60 | 51 | 2.88 | 1.33 |
| 3 | miR-423-5p/miR-378a-3p | LAC+Benign | 85 | 1.80 | 0.90 | 76 | 2.91 | 1.08 |
| 4 | miR-423-5p/miR-378a-3p | Control | 29 | 0.19 | 1.16 | 51 | 1.05 | 1.64 |
| 5 | miR-22-3p/sno-U57 | LAC+Benign | 85 | 6.94 | 1.07 | 76 | 3.90 | 2.02 |
| 6 | miR-22-3p/sno-U57 | Control | 29 | 4.63 | 0.85 | 51 | 1.70 | 1.61 |
| 7 | miR-126-5p/sno-U57 | LAC+Benign | 85 | 9.57 | 1.24 | 76 | 6.04 | 2.02 |
| 8 | miR-126-5p/sno-U57 | Control | 29 | 6.26 | 0.94 | 51 | 4.45 | 2.17 |
| 9 | miR-152-3p/sno-U57 | LAC+Benign | 85 | 4.02 | 1.52 | 76 | 0.18 | 2.13 |
| 10 | miR-152-3p/sno-U57 | Control | 29 | 0.65 | 1.03 | 51 | -1.46 | 1.96 |
| 11 | miR-423-5p/sno-U57 | LAC+Benign | 85 | 5.11 | 1.35 | 76 | 2.56 | 1.89 |
| 12 | miR-423-5p/sno-U57 | Control | 29 | 2.91 | 1.08 | 51 | -0.14 | 1.92 |
| 13 | miR-22-3p/sno-SNORD119 | LAC+Benign | 85 | 8.00 | 1.40 | 76 | 8.72 | 2.07 |
| 14 | miR-22-3p/sno-SNORD119 | Control | 29 | 5.11 | 1.60 | 51 | 6.48 | 2.05 |
|  | **LAC vs. Control** |  |  |  |  |  |  |  |
| 1 | miR-22-3p/miR-378a-3p | LAC | 50 | 3.99 | 0.57 | 44 | 4.18 | 1.14 |
| 2 | miR-22-3p/miR-378a-3p | Control | 29 | 1.80 | 0.60 | 51 | 2.88 | 1.33 |
| 3 | miR-423-5p/miR-378a-3p | LAC | 50 | 1.98 | 0.77 | 44 | 2.99 | 1.18 |
| 4 | miR-423-5p/miR-378a-3p | Control | 29 | 0.19 | 1.16 | 51 | 1.05 | 1.64 |
| 5 | miR-22-3p/sno-U57 | LAC | 50 | 6.98 | 1.09 | 44 | 3.54 | 1.83 |
| 6 | miR-22-3p/sno-U57 | Control | 29 | 4.63 | 0.85 | 51 | 1.70 | 1.61 |
| 7 | miR-126-5p/sno-U57 | LAC | 50 | 9.57 | 1.30 | 44 | 5.69 | 1.88 |
| 8 | miR-126-5p/sno-U57 | Control | 29 | 6.26 | 0.94 | 51 | 4.45 | 2.17 |
| 9 | miR-152-3p/sno-U57 | LAC | 50 | 3.84 | 1.46 | 44 | -0.06 | 1.91 |
| 10 | miR-152-3p/sno-U57 | Control | 29 | 0.65 | 1.03 | 51 | -1.46 | 1.96 |
| 11 | miR-423-5p/sno-U57 | LAC | 50 | 5.09 | 1.17 | 44 | 2.34 | 1.63 |
| 12 | miR-423-5p/sno-U57 | Control | 29 | 2.91 | 1.08 | 51 | -0.14 | 1.92 |
| 13 | miR-22-3p/sno-SNORD119 | LAC | 50 | 8.18 | 1.35 | 44 | 8.51 | 2.36 |
| 14 | miR-22-3p/sno-SNORD119 | Control | 29 | 5.11 | 1.60 | 51 | 6.48 | 2.05 |
|  | **LAC vs. Benign** |  |  | 0.754 |  |  |  | 0.742 |
| 1 | miR-374a-5p/miR-126-5p | LAC | 50 | -11.81 | 1.36 | 44 | -11.88 | 1.47 |
| 2 | miR-374a-5p/miR-126-5p | Benign | 35 | -12.74 | 1.72 | 32 | -12.86 | 1.05 |
| 3 | miR-374a-5p/miR-152-3p | LAC | 50 | -6.09 | 1.42 | 44 | -6.14 | 1.59 |
| 4 | miR-374a-5p/miR-152-3p | Benign | 35 | -7.43 | 2.05 | 32 | -6.84 | 1.01 |
| 5 | miR-374a-5p/miR-378a-3p | LAC | 50 | -5.36 | 1.66 | 44 | -5.55 | 1.71 |
| 6 | miR-374a-5p/miR-378a-3p | Benign | 35 | -6.76 | 2.12 | 32 | -6.37 | 1.53 |
| 7 | miR-374a-5p/miR-423-5p | LAC | 50 | -7.34 | 1.74 | 44 | -8.54 | 1.39 |
| 8 | miR-374a-5p/miR-423-5p | Benign | 35 | -8.29 | 2.11 | 32 | -9.19 | 1.27 |
| 9 | miR-374a-5p/tRNA-Thr-ACG | LAC | 50 | -8.53 | 2.10 | 44 | -5.72 | 1.77 |
| 10 | miR-374a-5p/tRNA-Thr-ACG | Benign | 35 | -9.47 | 1.56 | 32 | -6.64 | 1.56 |

LAC: lung adenocarcinoma; SD: standard deviation.

**Supplementary Figure S1.** Scatter plots comparing the expression ratios of the seven small ncRNA pairs in Panel 1 between the LAC+benign group and the control group for the training and validation stages.


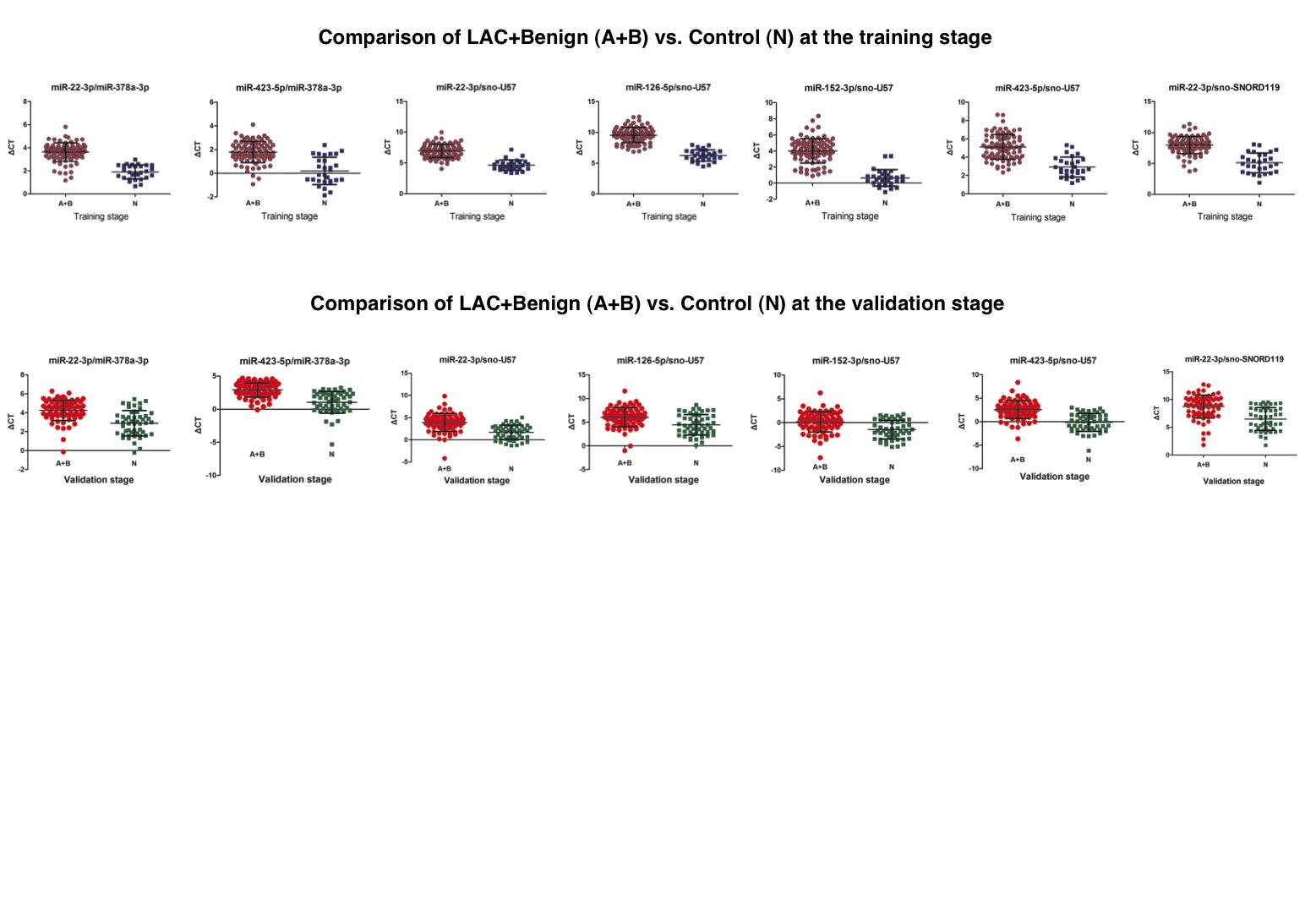


**Supplementary Figure S2.** Scatter plots comparing the expression ratios of the seven small ncRNA pairs in Panel 1 between the LAC group and the control group for the training and validation stages.


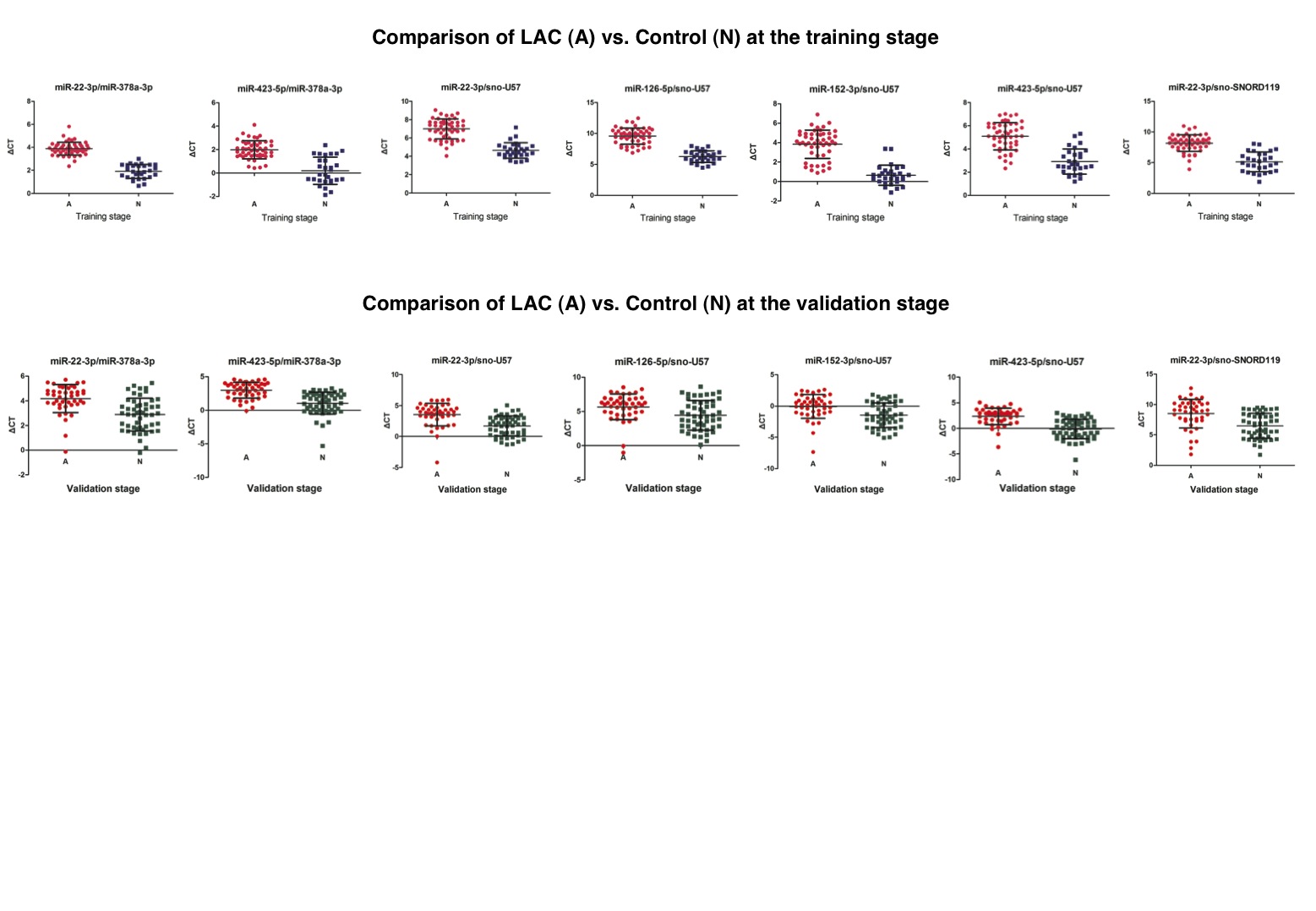


**Supplementary Figure S3.** Scatter plots comparing the expression ratios of the five small ncRNA pairs in Panel 2 between the LAC group and the benign group for the training and validation stages.


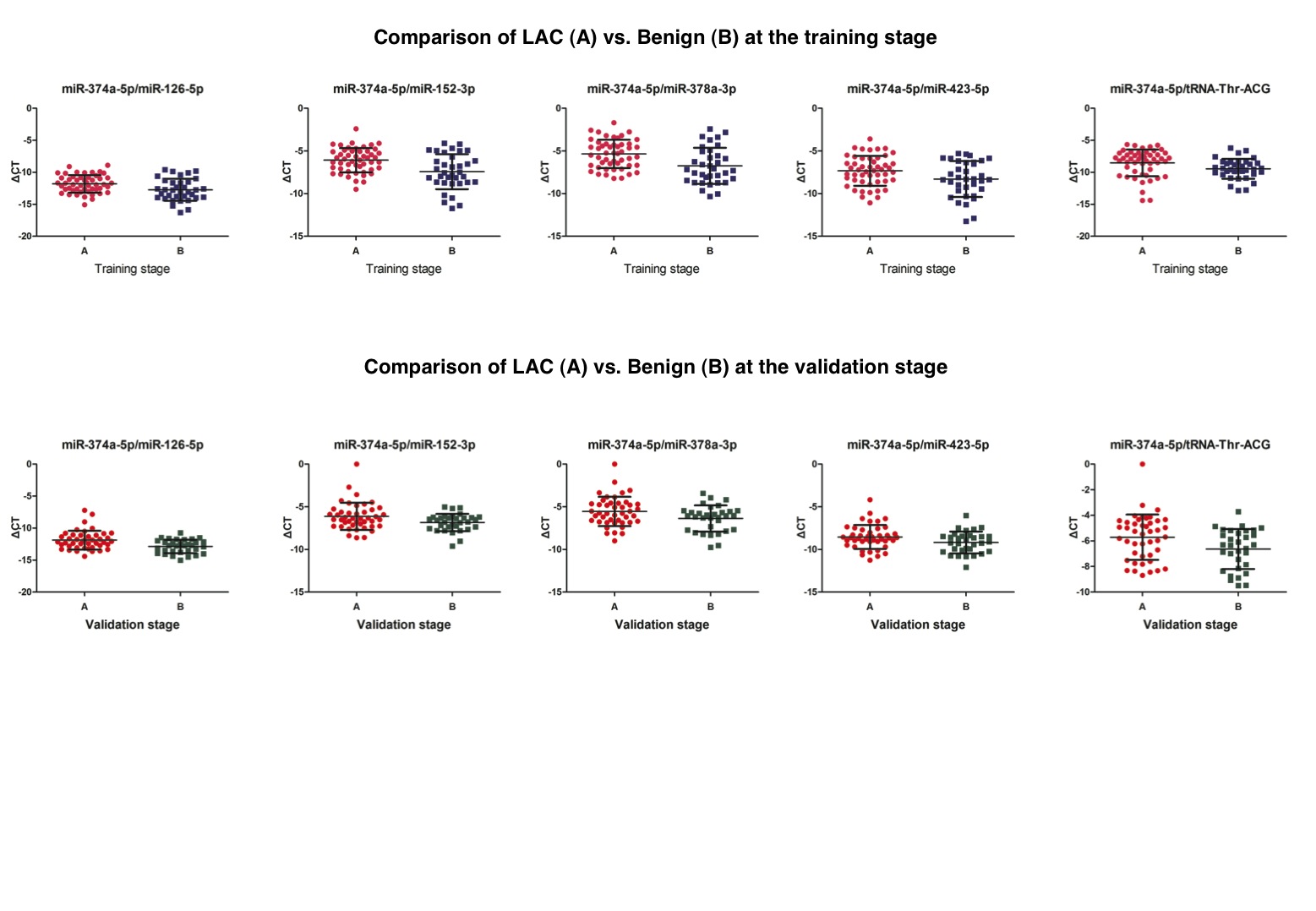

Supplement: Supplementary file 1 — Table S1. All candidate ncRNA pairs for lung cancer prediction. Table S2. Mean and standard deviation values of the expression ratios of the various ncRNA pairs for each group. Figure S1. Scatter plots comparing the expression ratios of the seven small ncRNA pairs in Panel 1 between the LAC+benign group and the control group for the training and validation stages. Figure S2. Scatter plots comparing the expression ratios of the seven small ncRNA pairs in Panel 1 between the LAC group and the control group for the training and validation stages. Figure S3. Scatter plots comparing the expression ratios of the five small ncRNA pairs in Panel 2 between the LAC group and the benign group for the training and validation stages. (DOCX 557 kb) [file 12864_2018_4862_MOESM1_ESM.docx]
